# Association between Parent-Child Relationship and Second-Time Mother’s Prenatal Depressive Symptoms: The Mediation Role of Parenting Burnout

**DOI:** 10.3390/ijerph20010491

**Published:** 2022-12-28

**Authors:** Ran Zhuo, Xiaoxue Shi, Ying Wu

**Affiliations:** College of Humanities & College of Home Economics, Jilin Agricultural University, Changchun 130118, China

**Keywords:** second-time mother, prenatal depressive symptoms, parent-child relationship, parenting burnout

## Abstract

Purpose: The aim of this research was to study the association between the mother-firstborn relationship and second-time mothers’ prenatal depressive symptoms before the birth of a second child and the mediation role of parenting burnout on this relationship. Methods: Empirical study was adopted in this research. Using a convenient sampling method, we recruited 110 second-time mothers who were in their third trimester of pregnancy. Child-parent relationship questionnaire, parenting burnout scale, and Beck Depression Instrument were used to measure the relationship between firstborn and second-time mothers, mothers’ parenting burnout, and prenatal depressive symptoms, respectively. Regression analysis was conducted to test the relationship between variables, and the mediation effect was tested using PROCESS. Results: Regression results showed that the parent-child relationship is negatively associated with second-time mothers’ prenatal depressive symptoms. The parent-child relationship is negatively associated with parenting burnout which is positively related to prenatal depressive symptoms. When considering the mediation variable of parenting burnout, the direct effect is not statistically significant. Conclusions: Parent-child relationship has a significant impact on second-time mothers’ prenatal depressive symptoms, and this relationship is mediated by parenting burnout.

## 1. Introduction

Prenatal depressive symptoms are a mood disorder that can affect women during pregnancy [1] with a wide range of symptoms, including hormonal shifts, physical ailments, sleep disruptions, and so on, affecting mental health directly (What are prenatal depressive symptoms? https://www.verywellmind.com/what-is-prenatal-depression-5240099, accessed on 12 November 2022). Second-time mothers, although they have experienced pregnancy of the firstborn, may have the same level of stress with their first pregnancy [2] with different stress sources. With the relaxation of the birth control policy in China, more and more families will have two or more children. Recent years have witnessed increasing numbers of transitioning from one-child families to two-child families. During the transition, everyone in the family must adjust their role to adapt to the changes. Parents, especially mothers, will have to spend more energy and time adjusting to a more complicated system, which undoubtedly increases mothers’ parenting pressure [3]. If the parenting pressure cannot be relieved, it can cause psychological problems like anxiety or depression [4]. Research has shown that prenatal depressive symptoms are increasing at an annual rate of 9% [5]. In particular, the incidence of depressive symptoms and anxiety in second-time mothers is significantly higher than that in first-time mothers. Therefore, second-time mothers’ depressive symptoms and their associated factors are of great importance in both academia and practice.

### 1.1. Parent-Child Relationship

The parent-child relationship is one of the pressure sources during the transition from a one-child family to a two-child family. Beyers-Carlso [6] analyzed 160 thousand posts on BabyCenter.com related to parenting problems from second-time mothers and found that whether the firstborn child can accept the second one and whether parents can treat the two children equally are the two themes discussed very often. J. Liu and Zhou [7] also showed that about one-third of firstborns were not supportive or neutral toward the coming siblings before birth. Their attitude has a significant impact on mothers’ well-being [7]. This phenomenon is very common in China because the long-time one-child policy made firstborns treated as little emperors in their families. They were the center of their families before, but the coming of a second baby would steal their thunder, which may generate jealousy. Children’s jealousy is a kind of social emotion, thoughts, and actions occurring in the family triangle (firstborn-sibling-mother) [8]. Complex jealousy makes the first child feel sad, painful, and angry. Meanwhile, jealousy will also cause behavior disorders in parent-child interaction or sibling interaction. The firstborns may attack their mother due to less attention from their mother, which may cause parent-child conflict, or they use some explicit behavior to attract their mothers’ attention [9,10]. Some studies have investigated the parent-child interaction after the birth of a second child [10]. They showed that after the birth of the second child, sibling rivalry would happen in the triangle. The one who fails in the competition will be jealous, which will lead to an imbalance of parent-child interaction. In addition, the firstborn’s problem behaviors and unsafe attachment will increase after the birth of a second child [11]. These factors are not conducive to the development of a good parent-child relationship. However, the impact of the transition on the parents, firstborn, and even the whole family starts when the mother gets pregnant. It has been shown that the firstborn has more problem behaviors, less emotional communication with their mother, and less safe attachment after their mother’s pregnancy [12,13]. In addition, second-time mothers’ emotional fluctuation increases due to the increase of cortisol hormone, making them more sensitive to their indocility behavior [14,15], which will highly likely cause conflict. The conflict may make the pregnant mother more stressed, which may lead to depression. Therefore, the parent-child relationship may impact second-time mothers’ prenatal depressive symptoms, but it received little attention in the literature. Therefore, this study makes the following hypothesis:

**Hypothesis** **1.** *Parent-child relationship negatively affects second-time mothers’ prenatal depressive symptoms.*

### 1.2. Parenting Burnout and Second-Time Mother’s Prenatal Depressive Symptoms

Parenting burnout is an emotional disorder in the context of parenting, which has three dimensions, including feelings of exhaustion in parenting, emotional distancing from one’s children, and loss of pleasure and efficacy in the parental role [16]. Parenting burnout does not only impact children’s development but also affects mother’s mental health [17,18,19]. With the increase in distance between parent and child because of pregnancy, the risk of parenting burnout may also increase [20]. The risk-balance theory in parenting burnout shows that when parenting pressure (risk factor) is high, individuals must use more resources (protective factor) to cope with it [16]. If the resources cannot meet parenting demands for a long time, exhaustion will occur psychologically. Therefore, we propose the following hypothesis:

**Hypothesis** **2.** *Parenting burnout has a positive impact on second-time mothers’ prenatal depressive symptoms.*

### 1.3. Parent-Child Relationship and Parenting Burnout

The parent-child relationship does not only affect the mother’s mental health but also has an impact on parenting pressure [21]. The parent-child relationship is developed through vertical interactions in a family with symmetry and complementary characteristics [22]. During the interaction, parents can impact children’s development of behavior and cognition. Meanwhile, parents’ health can also be impacted by children [23]. As a result, the interaction between parents and children not only affects children’s development but also affects mothers’ health. Moïra Mikolajczak [24] showed that family relationships and family cohesiveness could significantly predict parenting burnout. However, it didn’t study the impact of the parent-child relationship on parenting burnout. J.D. DeFreese [25] demonstrated that harmonized parent-child relationships in sports could positively predict burnout. The impact of the parent-child relationship on parenting burnout can be argued from two aspects. On the one hand, a good parent-child relationship means more interaction and more intimacy between the child and the mother. The mother may get more positive feedback from her child and would enjoy more of the parenting process. Children’s positive emotional response (like a smile or hugging) is a very important positive psychological resource according to the risk and resource balance theory of parenting burnout [16]. Therefore, a good parent-child relationship is a family factor that may decrease a mother’s parenting burnout. With a good relationship, mothers can feel happiness in parenting and feel the meaning of life [22]. When parents encounter psychological problems, a good parent-child relationship can provide more support [26], which decreases parenting pressure). On the other hand, a poor parent-child relationship may increase children’s risk-taking behavior and problem behavior [7], which increases the difficulty of parenting.Dai showed that intimacy and conflict in a parent-child relationship have a significant correlation with parenting pressure [27]. Therefore, we propose the following hypothesis:

**Hypothesis** **3.** *Parent-child relationship has a negative impact on parenting burnout.*

**Figure 1:**
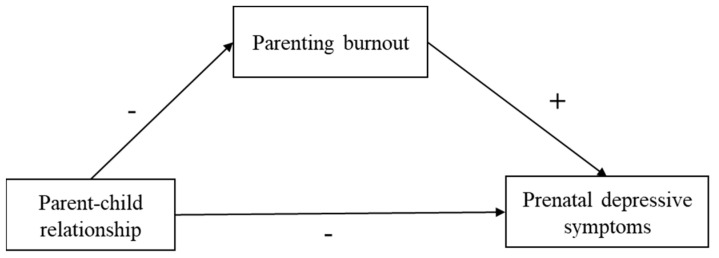
Conceptual Model. Note: “+” means positive forecast, and “−“ means negative forecast.

To sum up, this study aimed to examine the relationship between the parent-child relationship and prenatal depressive symptoms of second-time mothers during pregnancy, as well as the mediating role of parenting burnout between them. Based on the above hypotheses, we developed a conceptual model, as shown in Figure 1.

## 2. Participants and Methods

### 2.1. Participants

We recruited second-time mothers as our participants who should satisfy the following criteria: (1) Firstborns, and their mothers do not have any physical health issues; (2) Mothers are in the third trimester of their pregnancy. The research was approved by the authors’ Research Ethics Committee. One hundred ten participants were recruited via social media Wechat using a convenient sampling method between March 2019 and September 2021. Based on Green’s [28] rule of thumb, 107 samples are required for testing the coefficients in linear regression with a significance level of 0.05, 3 predictors, 0.8 statistical power, and medium effect. Therefore, our 110 samples are sufficient for valid statistical analysis. 

The participants were asked to fill out an online questionnaire with the support of our research assistants. All the 110 returned questionnaires were valid. The sample came from 20 provinces in China, with Jilin Province accounting for 37.14% and others between 1% to 9%. Among the 110 participants, the firstborns’ average age was 84 months (M = 7.24, SD = 2.67), and second-time mothers were aged between 24 and 41, with an average age of 33 (M = 32.83, SD = 3.75). The average marriage time of the participants and their husbands was 8 years (M = 7.97, SD = 3.04), and they all lived with their first child. The demographic information of the sample is listed in Table 1. From Table 1, we can see that more than 59% of mothers received higher education. About half of families’ annual income was between 10–50 thousand RMB Yuan, and 43% of families’ annual income was lower than 10 thousand RMB Yuan. This means that our sample represents a well-educated middle- and lower-income population.

### 2.2. Measurements

#### 2.2.1. Child-Parent Relationship Questionnaire

The Child-Parent relationship questionnaire was developed by Pianta and Virginia [29] and has been validated in the Chinese context [30]. The questionnaire consisted of 30 items, including three dimensions of intimacy, dependency, and conflict. It was completed by mothers or fathers to measure parents’ perceptions of their relationship with their child, suitable for 2–6 years old children. A 5-point Likert scale was used from 1 (definitely does not apply) to 5 (definitely applies) to describe the applicability degree between the statements and the father/mother’s perception. The higher the score, the better the parent-child relationship. In our sample, Cronbach’s α coefficients for intimacy, conflict, and dependency were 0.78, 0.89, and 0.58, respectively.

#### 2.2.2. Parenting Burnout Scale

The parenting burnout scale was developed by Le Vigouroux [31] and validated in the Chinese population by Zou [30] This scale had 22 items, assessing emotional exhaustion, emotional distancing, parental accomplishment, and efficacy. It used a 7-point Likert scale from 1 (never) to 7 (every day). The higher the score, the higher the parenting burnout. In this sample, Cronbach’s α coefficient was 0.92.

#### 2.2.3. Prenatal Depressive Symptoms

The Beck Depression Instrument (BDI) [32] was used to measure second-time mothers’ prenatal depressive symptoms. The Chinese version has shown a very high internal reliability and current validity [33]. The BDI has 21 items, which have been widely used in different research, including prenatal depressive symptoms research [34]. The score was from 0 to 3. The total score was used by summing up the score of each item. In this sample, Cronbach’s α coefficient was 0.90.

## 3. Results

### 3.1. Data Screening

Before conducting the data analysis, we first checked the data so that they met the requirements of the statistical methods. All the questionnaires were valid, and there were no missing data or outliers because of the research assistants’ support during the process of completing the questionnaire. To check the normality of the variables, we calculated the kurtosis and skewness of the data as follows: parenting burnout (1.64, 2.72) and prenatal depression (1.59, 4.02). Considering the sample size was not too big, we used Shapiro–Wilk normality tests, and the result was as follows: parenting burnout (*p* = 0.00) and prenatal depression (*p* = 0.00). Therefore, we can say that the data followed a normal distribution. In addition, we used Harman’s single-factor test to check the common method bias. It showed that there were 74 factors whose eigenvalues were greater than 1, and the first factor explained 24.10% cumulated variance, which is lower than 40%. Therefore, it can be claimed that there was no serious common method bias [35]. In summary, the data were suitable for statistical analysis.

### 3.2. Data Analysis

#### 3.2.1. Descriptive Statistics

We first report the descriptive statistics of the scales, which are presented in Table 2. 

#### 3.2.2. Correlation Analysis

We first conducted a correlation analysis, which is shown in Table 2. We can see that the parent-child relationship was negatively correlated with the mother’s prenatal depressive symptoms. The parent-child relationship was positively correlated with parenting burnout, and so were the parenting burnout and prenatal depressive symptoms. A mother’s education level was negatively correlated with prenatal depressive symptoms. So, we can proceed to conduct regression analysis to see the relationship between the variables. 

#### 3.2.3. Main Effect Analysis

To test hypothesis 1, that is, the parent-child relationship can negatively predict prenatal depressive symptoms of second-time mothers, linear regression analysis was used. A mother’s prenatal depressive symptoms were used as the dependent variable and the parent-child relationship as the independent variable. The mother’s education level was entered into the regression function as a control variable. The result is presented in Table 3. As shown in Table 3, the parent-child relationship can significantly negatively predict second-time mothers’ prenatal depressive symptoms after excluding the mixed interference of the mother’s education level, and its coefficient value is −0.395. This result shows that during the transition period, the closer the parent-child relationship, the better the mother’s health. Hypothesis 1 was supported. 

#### 3.2.4. Mediation Effect Analysis

**Figure 2:**
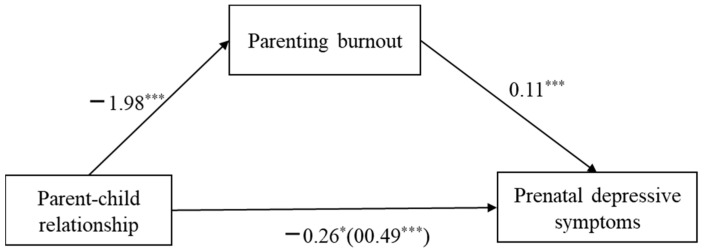
Parenting burnout mediated the relationship between parent-child relationship and second-time mothers’ prenatal depressive symptoms with standardized coefficients. * *p* < 0.05, *** *p* < 0.001.

To further test the mediation effect of parenting burnout between parent-child relationship and prenatal depressive symptoms, i.e., Hypotheses 2 and 3, we used the SPSS macro PROCESS. A mother’s education level entered the model as a control variable. As shown in Table 4, the parent-child relationship can significantly predict second-time mothers’ parenting burnout (*a* =−1.98, *SE* = 0.32, *p* < 0.001). Parenting burnout can significantly predict prenatal depressive symptoms (*b* = 0.12, *SE* = 0.03, *p* < 0.001). The parent-child relationship can significantly predict prenatal depressive symptoms (*c* =−0.26, *SE* = 0.12, *p* < 0.05). Therefore, Hypotheses 2 and 3 were supported. This result is depicted in Figure 2. It was shown that parenting burnout could mediate the relationship between the parent-child and prenatal depressive symptoms partially. The standardized mediation effect is (1.98) × (0.12) = 0.23. We used a bootstrapping procedure to test the significance of the indirect effect with 5000 bootstrapped samples. The results are presented in Table 5. 

## 4. Discussion

### 4.1. Parent-Child Relationship’s Impact on Second-Time Mothers’ Prenatal Depressive Symptoms

Our results have shown that the parent-child relationship can significantly predict second-time mothers’ prenatal depressive symptoms. This result is consistent with the literature on the association between the parent-child relationship and the mother’s mental health [21,36,37]. Considering the parenting culture difference between western countries and China and the recent changes in childbirth policy in China, second-time mothers’ prenatal depressive symptoms are of great importance in a China context. Our research contributed to the literature by using Chinese second-time mothers as a sample and showed that the parent-child relationship has a significant impact on a mother’s mental health during the transition from one child to siblings. The change in family structure will certainly affect the interaction mode of family members [38]. The research on the interaction between the first child and the mother within six months of the second child’s birth showed that 46.8% of the mothers felt an increase in parent-child conflict [39]. Other studies have also proved that the quality of interaction between the first child and the mother declines with the birth of the second child. For example, aggressive behavior would increase, emotional reactions to the mother would decrease, and hugging times would be less [40,41]. It was also demonstrated in a China context that the interaction between the prenatal mother and the first child could affect the mother’s attachment to the second child [42,43]. Different from the existing results, our research showed that the firstborns’ interactions with their mothers could impact their mothers’ mental health before the birth of the second child. Some studies in China also indirectly support our results. J. Liu [7] showed that the first child’s acceptance of the second one is significantly related to the health of the mother. Even though this research did not directly indicate the relationship between the parent-child relationship and the health of the second-time mother, the attitude of non-acceptance of the first child indicates the formation of the competition triangle (sibling–mother). At the same time, in this relationship structure, the firstborn will also make behaviors and emotional reactions to fight for the position [9,10]. The behavior disorder or reduced safe attachment will inevitably increase the difficulty of the mother’s parenting.

Family theory and ecological system theory [44,45] state that an individual’s psychological development depends on their interactions with the environment of the family system. The firstborn, as an important member of the family system, is an important impact factor in the mother’s mental health. Abidin’s parenting pressure modes also support our research [46]. Parents’ parenting pressures are affected by parents’ personalities, disordered parent-child interaction, and children’s behavior. Thus, a poor relationship between parent and child is one of the key factors which could result in parenting pressure [46]. If there exists a developmental risk in the parent-child relationship, mothers will face more parenting pressures, which will cause health issues for mothers. With the increasing parenting pressure, parents’ emotional disturbance would be worse [47](, and depression is very likely to happen.

### 4.2. The Mediation Role of Parenting Burnout

Our research has shown that parenting burnout played a mediation role, meaning that the parent-child relationship impacts the mothers’ prenatal depressive symptoms through second-time mothers’ parenting burnout. This result is consistent with the risk-balance theory in parenting burnout. The poor parent-child relationship brings more challenges to the mother’s parenting. When mothers cannot fulfill the parenting tasks, they will feel emotional exhaustion. On the contrary, a good parent-child relationship reduces the mother’s parenting burnout. Different from the existing research, our research focused on burnout in the parenting environment. Parenting does not only include doing sports with firstborns but also includes study guidance, moral education, accompanying, etc. Although there is little research on the impact of the parent-child relationship on parenting burnout in the literature, there is some research supporting our results indirectly. Vigouroux [20] research on the impact of children’s propensity factor on mothers’ parenting burnout showed that children who have stable emotions are easy to get along with their parents and can negatively predict mothers’ parenting burnout. Lebert-Charron [48] also showed that attachment avoidance is a risk factor for parenting burnout. Based on Gillis [49], our research further demonstrated that a poor parent-child relationship could result in parenting burnout, while a good parent-child relationship can positively affect parenting burnout. In addition, parenting burnout could lead to second-time mothers’ prenatal depressive symptoms. Due to parenting burnout with the firstborn, second-time mothers may feel exhausted, which could result in pressure and depression. Therefore, the impact of the parent-child relationship can affect second-time mothers’ prenatal depressive symptoms through parenting burnout. 

### 4.3. Generalizability of the Results

As mentioned in Section 2, our samples were from a well-educated middle- and lower-income population. Although it was a large group of modern Chinese women, it cannot represent all second-time mothers. Therefore, we should be cautious about generalizing the results of this study to all second-time mothers in China. In addition, due to the cultural differences between China and other countries, the results may not apply in other cultural contexts. Cross-cultural comparative studies could be interesting.

## 5. Conclusions

This study contributed to the study of influencing factors of second-time mothers’ prenatal depressive symptoms. Most of the studies only focused on the impact of the parent-child relationship on children and did not distinguish between first-time or second-time mothers. This study was the first to show that the parent-child relationship can predict second-time mothers’ prenatal depressive symptoms through the influence of parenting burnout. In addition, the samples of this study were all from China. Because of the long-term birth control policy, the firstborn has always been the little emperor of the family. They may be more sensitive to the arrival of their siblings, and their behavioral and emotional reactions may be more intense and last longer. The interaction between the first child and the mother may have a greater impact on the mother’s prenatal depressive symptoms. Therefore, this result will contribute to the research in the field of second-child family transition. This result suggests that we should also pay attention to the first child for the prevention of prenatal depressive symptoms of the second-time mother, and the family should pay attention to and respond to the first child in time to help the firstborns adapt to the new family structure. The government should also provide some family intervention services for families before the birth of a second child to prevent psychological discomfort between the first child and the mother during the transition period.

## 6. Limitations and Future Research

This study is not without limitations. First, a second-time mother’s mental health history may have an impact on their psychological status during their pregnancy. Therefore, including their mental health history in the model will be a future research direction. Second, the parent-child relationship in this study only involves the relationship between the mother and the first child. Further research should include the influence of the father’s reported parent-child relationship on the mother’s prenatal depressive symptoms. Because fathers also play an important role in childcare, and a mother’s energy and physical strength may be poor during pregnancy, so the firstborns may rely more on the father’s emotional and behavioral support. Research has proved that the father-son (daughter) relationship can actively help the firstborn adapt to the new family structure [3]. In addition, we need to further use cross-sectional research to further determine the causal relationship between variables and the change paths of the parent-child relationship and prenatal depressive symptoms during pregnancy of second-time mothers. Moreover, part of our data was collected during the COVID-19 pandemic, but the impact of COVID-19 on pregnant women was not considered. Research has shown that COVID-19 may have different effects on pregnant women [50]. Future research should take the impact of COVID-19 into account. Finally, we do not have information about the parent-child relationship before the mother became pregnant, so we cannot determine the independent impact of the second child transition on the parent-child relationship.

## Figures and Tables

**Table 1:** Demographic information of the sample (*N* = 110).

	Value	Number	Percentage
Firstborns’ gender	boy	47	42.7%
	girl	63	57.3%
Family annual income	Below 100 k	48	43.6%
	100 k–200 k	42	38.2%
	200 k–500 k	14	12.7%
	Above 500 k	6	5.5%
Maternal education	Below undergraduate	51	46.4%
	Undergraduate or above	59	53.6%
Working status	Full-time work	64	58.2%
	Part-time work	12	11.9%
	No work	34	30.9%
Firstborn’s main carer	Myself	57	51.8%
	My husband	2	1.8%
	My husband’s parent/s	28	25.5%
	My parent/s	19	17.3%
	Babysitter	4	3.6%
	Other	0	0%

**Table 2:** Correlation analysis.

	Mean	SD	Parenting Burnout	Prenatal Depressive Symptoms	Parent-Child Relationship
Parenting burnout	2.15	1.17			
Prenatal depressive symptoms	1.51	0.405	0.469 **		
Parent-child relationship	3.41	0.32	−0.544 **	−0.422 **	
Mother’s education level	1.53	0.50	−0.105	−0.219 *	0.194 *

* *p* < 0.05, ** *p* < 0.01.

**Table 3:** Main effect.

	*B*	*SE*	*β*	*p*
Constant	3.394	0.378		0.000
Mother’s education level (Lower than undergraduate)	−0.115	0.071	−0.142	0.110
Parent-child relationship	−0.499	0.112	−0.395 ***	0.000
*R* ^2^	0.198			

*** *p* < 0.001.

**Table 4:** The regression result with a mediator.

Dependent Variable	Independent Variable	*R*²	*F*	*β*	95% *CI*	
					*Lower Limit*	*Upper Limit*
Parenting burnout	Mother’s education (Lower than undergraduate)	0.29	22.44 ***	0.00	−0.38	0.38
	Parent-child relationship			−1.98 ***	−2.58	−1.38
Prenatal depressive symptoms	Mother’s education (Lower than undergraduate)	0.27	13.68 ***	−0.11	−0.25	0.01
	Parenting burnout			0.1177 ***	0.05	0.18
	Parent-child relationship			−0.26 *	−0.51	−0.01

* *p* < 0.05, *** *p* < 0.001.

**Table 5:** Effect values.

	*Effect*	*SE*	95% *CI*	
			Lower *CI*	Upper *CI*
Total effect	−0.49	0.11	−0.72	−0.27
Direct effect	−0.26	0.12	−0.51	−0.01
Indirect effect	−0.23	0.12	−0.49	−0.01
Standardized indirect effect	−0.57	0.26	−1.08	−0.03

## Data Availability

The dataset in this research can be obtained upon request.
